# Ethnic impact on knee osteoarthritis pain predictors in an urban Malaysian population: a retrospective study

**DOI:** 10.7717/peerj.20911

**Published:** 2026-03-13

**Authors:** Mohd Azzuan Ahmad, Mohamad Shariff A. Hamid, Ashril Yusof

**Affiliations:** 1 Physiotherapy Programme, Centre for Rehabilitation and Special Needs Studies, Faculty of Health Sciences, Universiti Kebangsaan Malaysia, Kuala Lumpur Federal Territory, Malaysia; 2 Sports Medicine Unit, Faculty of Medicine, Universiti Malaya Medical Centre, University of Malaya, Kuala Lumpur Federal Territory, Malaysia; 3 Faculty of Sports and Exercise Science, University of Malaya, Kuala Lumpur Federal Territory, Malaysia

**Keywords:** Alcohol consumption, Cigarette smoking, Ethnicity, Knee osteoarthritis, Prevalence, Sociodemographic, Urban population

## Abstract

**Objective:**

Knee osteoarthritis (KOA) is a degenerative joint disease associated with chronic pain and disability. Its epidemiology in Malaysia’s diverse urban population remains unclear, as previous studies often relied on self-reports, non-standardized diagnostic criteria, and predominantly rural samples. This study assesses the prevalence of symptomatic KOA and its sociodemographic and pain-related predictors in an urban Malaysian cohort.

**Methods:**

This retrospective cross-sectional study drew data from newly diagnosed cases of symptomatic KOA at University Malaya Medical Centre, based on the American College of Rheumatology criteria, between 2017 and 2020. A random, ethnicity-stratified sample of 600 medical records was analysed using descriptive and logistic regression statistics.

**Results:**

Of 17,253 new cases, 20.5% (*n* = 3,529) were diagnosed with symptomatic KOA; (i) 43.4% were Chinese, 28.9% Malay, 27.6% Indian, and 2.1% from other ethnic groups. Most patients were aged ≥65 (60.2%), female (72.1%), and overweight/obese (71.1%). Significant associations were observed between symptomatic KOA prevalence and (i) alcohol consumption among Chinese (62.9%) and (ii) low financial status, smoking, and physical inactivity among Indians (33.3%). Modifiable predictors of poor pain outcomes included sedentary lifestyles and non-adherence to rehabilitation, while severe disease stage and lower education were non-modifiable predictors.

**Conclusion:**

Ethnic disparities in KOA prevalence and risk factors in urban Malaysians highlight the importance for tailored interventions to improve rehabilitation adherence, promote physical activity, and address lifestyle risks such as alcohol and smoking. These findings emphasize the need for culturally sensitive healthcare strategies to ensure effective KOA management in this diverse population.

## Introduction

Knee osteoarthritis (KOA) is a degenerative joint pathology characterized by progressive structural deterioration, including cartilage degradation, subchondral bone changes (Chin, Ekeuku & Pang, 2022; Hayashi, Roemer & Guermazi, 2025), synovial inflammation, and biomarker alterations (Jenei-Lanzl & Zaucke, 2025). These pathological changes are often accompanied by significant functional impairments, chronic pain, and disability (Ahmad et al., 2018; Courties et al., 2024). The global prevalence of KOA is increasing, with approximately 654 million people diagnosed with KOA in 2020 (Cui et al., 2020). Research suggests that the prevalence of KOA may differ depending on geographical location, ethnicity, and culture (Courties et al., 2024; Cui et al., 2020). Within the multi-ethnic Malaysian population, influenced by the diverse Malay, Chinese, and Indian cultures, current research is hindered by the absence of standardized KOA diagnoses, such as those defined by the American College of Rheumatology (ACR) criteria (Chia et al., 2016; Mat et al., 2019). Instead, diagnoses are often reliant on patients’ self-reported symptoms, leading to imprecise data on the actual prevalence of KOA. Furthermore, there is a lack of information regarding the sociodemographic factors associated with KOA, particularly with respect to ethnic disparities in the urban Malaysian setting (Chia et al., 2016; Mat et al., 2019). Given these gaps in knowledge, it becomes imperative to refocus research efforts on urban settings, where diverse populations and lifestyles converge.

Based on several studies in Malaysia, the prevalence of KOA is reported to be between 10 and 30% (Chia et al., 2016; Mat et al., 2019; Zakaria et al., 2009), and it predominantly affects females aged 50 years and above (Mat et al., 2019). Apart from the variance in KOA prevalence based on geographical population, there have also been ethnic differences reported (Callahan et al., 2021; Mat et al., 2019). An earlier study on the prevalence of KOA-related pain in Malaysia reported the highest rate among Indians and the lowest among Chinese (Chia et al., 2016). In contrast, a later study by Mat et al. (2019) reported that the prevalence of KOA in Malaysia was 31%, with Malays (45%) having the highest rate and Indians (32%) having the lowest. It is noted that these studies were analyses primarily based on unspecified self-reported symptoms (mostly knee pain) rather than definite clinical or radiographic KOA diagnoses according to the ACR criteria (Chia et al., 2016; Mat et al., 2019). Furthermore, it is also important to note that the data analyzed in these studies did not represent urban populations. The inclusion of rural populations may have introduced significant variability in the findings, as urban and rural areas often feature distinct geographical, lifestyle, and healthcare access factors that can influence disease prevalence and its effects (Ji et al., 2023). As a result, placing dedicated emphasis on urban settings in Malaysia is paramount.

Several factors are known to be associated with KOA, particularly (i) non-modifiable factors such as age ≥50 years, female sex (Courties et al., 2024), level of education (Callahan et al., 2021) and financial status (Ji et al., 2023; Lee et al., 2021) and (ii) modifiable factors: body mass index (BMI) (Mündermann et al., 2024; Zulfarina et al., 2022), cigarette smoking (Kong et al., 2017), alcohol consumption (Joseph et al., 2019; Szilagyi et al., 2023) and physical inactivity (Kraus et al., 2019). Most KOA management guidelines recommend altering identified modifiable risk factors through targeted interventions for optimal clinical outcomes (Lawford et al., 2024; Yeap et al., 2021); interestingly, these factors can be influenced by cultural and religious background (Callahan et al., 2021; Cui et al., 2020). For instance, Asians are predisposed to KOA due to their cultural routine (*e.g*., floor sitting, squatting for housework and toileting) which increases the repetitive load on the knee joint, especially among those who are overweight or obese (Ji et al., 2023; Sathiyamoorthy, Ali & Kloseck, 2018). Additionally, religious and sociocultural norms were reported to negatively affect alcohol consumption within Muslim societies (Alhashimi et al., 2018), primarily linked to Malay ethnicity as Muslim adherents (Arshad, Rozali & Kamaruzzaman, 2012).

Comprehensive epidemiological data regarding the KOA population in Malaysia remain notably scarce, particularly within urban contexts (Chia et al., 2016; Mat et al., 2019). Previous studies predominantly relied on self-reported criteria, primarily centered around knee pain, without specifying the underlying conditions responsible for the pain, such as muscle, ligament, or meniscus injuries (Chia et al., 2016; Mat et al., 2019). Additionally, these studies collected data from relatively small and homogenous samples, primarily consisting of older age groups, and often encompassing both urban and rural settings (Chia et al., 2016; Mat et al., 2019). Acknowledging the distinctive challenges presented by urban environments and the dynamic shifts in lifestyle and healthcare access patterns within such settings, this study endeavors to fill these critical knowledge gaps. Hence, this study aimed to (i) determine the prevalence and sociodemographic characteristics of patients with symptomatic KOA (based on the ACR criteria), (ii) examine factors associated with KOA-related outcomes, and (iii) identify predictors of KOA pain outcomes within the multi-ethnic urban population of Malaysia. The findings are expected to expand upon existing literature and identify sociodemographic factors that might predict high-risk groups, thereby facilitating the development of more targeted interventions for KOA within urban Malaysia.

## Methods

### Study design

This study employed a retrospective cross-sectional design, relying on secondary data obtained from the hospital database and patients’ medical records.

### Ethical approval

The study protocol received approval from the Medical Research Ethics Committee of the University Malaya Medical Centre (UMMC) (MREC ID: 2019124-8061), following the guidelines outlined in the Helsinki Declaration of 1975. Considering that this study involved only secondary data collection, no written consent was required.

### Study setting and population

The selection of UMMC as the research site was deliberate, as it functions as the primary referral hospital in the vibrant urban centre of the Klang Valley (Peng & Li, 2022). This area is inhabited by a diverse population of approximately nine million individuals, representing various ethnicities and constituting 30% of Malaysia’s total population (Peng & Li, 2022). Medical records of newly-diagnosed patients with symptomatic KOA at the UMMC Primary Care Clinics were reviewed. The inclusion criteria were (i) Malaysian adults aged 18 years or older, (ii) who visited any of the identified outpatient clinics between January 2017 and December 2020 as a new patients, and (iii) were newly diagnosed with unilateral or bilateral symptomatic KOA based on the ACR clinical or radiographic criteria. The ACR clinical criteria for KOA include the presence of knee pain with at least three of the following: age >50 years, morning stiffness lasting <30 min, crepitus on active motion, bony tenderness, bony enlargement, and the absence of palpable warmth of the joint (Altman et al., 1986; Zhang et al., 2010). Meanwhile, the exclusion criteria were follow-up cases and patients with KOA who had defaulted on treatment and returned as new patients. At UMMC, the diagnostic process for KOA is standardized across primary care, orthopedic, and rheumatology clinics. Attending physicians establish the diagnosis using the ACR criteria as per hospital guidelines, and the confirmed diagnosis is subsequently coded into the International Classification of Diseases, 10th Revision (ICD-10) system within the hospital’s electronic medical record. Thus, all cases captured in this study represent symptomatic patients who presented primarily with knee-related complaints and fulfilled the ACR diagnostic criteria, rather than being identified by ICD-10 codes alone. To ensure accuracy, the extracted list of ICD-10 coded cases was cross-validated through discussions with the Medical Records Department and verified by a consultant orthopaedic physician on the research team.

### Sample size

The required sample size for the medical records review was estimated using a formula by Daniel (1999), with recommended parameters based on Naing, Winn & Nordin (2006). A Z-value of 1.96 was used for a conventional 95% confidence level, with an expected prevalence (*P*) of 25% and a precision (*d*) of 0.05 (Naing, Winn & Nordin, 2006). The expected prevalence was set at 25% based on previous studies that reported prevalence rates of KOA ranging from 20 to 30% in the Malaysian population (Chia et al., 2016; Mat et al., 2019). The minimum sample size required was calculated to be 288 medical records.

${\rm Sample \; size}, {\rm n}= {{{Z^2}P\left( {1 - P} \right)} \over {{d^2}}}={{{{1.96}^2}\times 0.25\; \left( {1 - 0.25} \right)} \over {{{0.05}^2}}}=288.$

However, to ensure the robustness of the data and account for potential attrition due to missing or incomplete records, the sample size was increased to 600 medical records. These records were stratified evenly across the three major ethnic groups in Malaysia; Malay (*n* = 200), Chinese (*n* = 200), and Indian (*n* = 200). Stratification ensured adequate representation of each group and enabled subgroup analyses, reflecting Malaysia’s multi-ethnic population structure and supporting a more comprehensive understanding of KOA prevalence. These subgroup analyses were exploratory in nature, designed to provide additional insights into ethnic and sociodemographic differences. The main conclusions of this study were therefore based on the overall cohort, while subgroup findings were interpreted with caution due to potential limitations in statistical power.

### Procedures

The electronic master list of all new cases registered at the UMMC from January 2017 to December 2020 was obtained from the Medical Records Department. A total of 600 medical records were selected by stratified (ethnicity) sequential (odd numbers) randomization; Malay (*n* = 200), Chinese (*n* = 200), and Indian (*n* = 200). Patients’ sociodemographic and KOA-related data were extracted into the clinical research form (Appendix A). To ensure sample reliability, selected medical records with more than 10% missing data were excluded and replaced with new records.

### Data variables

The data collected were (i) sociodemographic information: age, sex, ethnicity, BMI, education background, financial status, smoking status, alcohol consumption, and physical activity level; (ii) disease-related variables, which encompassed mode of diagnosis (knee radiograph), severity of KOA using the Kellgren-Lawrence classification (Kellgren & Lawrence, 1957; Schiphof, Boers & Bierma-Zeinstra, 2008); and (iii) rehabilitation: physiotherapy referral, patients’ adherence to rehabilitation, and KOA pain outcomes (changes in knee pain scores from the time of diagnosis or first visit compared to follow-up assessments up to six months later). The K-L classification (Kellgren & Lawrence, 1957; Schiphof, Boers & Bierma-Zeinstra, 2008) recorded in the hospital system was assessed by the attending orthopedic physicians responsible for patient management. In cases where the grading was not recorded or unconfirmed, one orthopedic consultant from the research team independently evaluated the available records and imaging to determine the appropriate K-L grade. For the purpose of analysis, we grouped K–L grades 1 and 2 as mild, grade 3 as moderate, and grade 4 as severe. This approach is consistent with previous literature where K–L grades 1 and 2 are considered early or mild OA, grade 3 represents definite joint space narrowing and osteophyte formation indicative of moderate OA, and grade 4 reflects advanced disease with severe joint space narrowing and sclerosis (Kellgren & Lawrence, 1957; Schiphof, Boers & Bierma-Zeinstra, 2008).

To ensure consistency, data extraction for each case commenced from the date of the initial diagnosis visit and continued until the six-month follow-up, aligning with hospital rehabilitation management policies that aim to discharge KOA patients for self-management within this timeframe. In this study, age was categorized into three groups based on previously reported risk stratification: 18 to 44 years (young adults), 45 to 64 (middle-aged group), and ≥65 (elderly group) (Plotnikoff et al., 2015). Meanwhile, BMI was described based on the Asia-Pacific classification (Lim et al., 2017).

Patients’ education background was categorized as primary-secondary or tertiary. Financial status was classified based on whether patients required financial assistance (aided) or did not require financial assistance (unaided). This was determined through records of employment, occupation, and monthly income. Physical activity was assessed based on any related information noted in the medical records, where individuals were classified as active if they were recorded as participating in sports or had an active lifestyle; those described as sedentary or not participating in sports were classified as sedentary. Smoking status was recorded as current smoker, former smoker, or non-smoker. Alcohol consumption was categorized into current drinkers and non-drinkers, with current drinkers further differentiated into moderate or heavy drinkers if the data permitted. Moderate drinking was defined as consumption of up to seven drinks per week, whereas heavy drinking was defined as alcohol intake exceeding this threshold (Mutalip et al., 2014; World Health Organization, 2018).

Patients’ adherence to their rehabilitation program was categorized as adhered (attending ≥50% of sessions and complying with the home exercise program) or non-adhered (attending <50% of sessions, not complying with the home exercise program, or withdrawing from the rehabilitation program). Adherence was assessed through detailed physiotherapy notes. Any missed sessions were recorded as absence by the therapist, as follow-up sessions were pre-scheduled. During re-evaluation, therapists also inquired about the patient’s compliance with the prescribed home exercise program, which was also documented. KOA pain outcomes were extracted from the reported knee pain scores, primarily through the visual analogue scales (VAS) or numerical pain rating score (NPRS). Changes in pain scores were calculated by comparing the initial VAS or NPRS scores from the first visit with the last available scores at follow-up, regardless of the number of follow-ups, due to variability in the data.

### Statistical analysis

Data were analyzed using SPSS version 25.0 (IBM, Armonk, NY, USA). Based on the study objectives, descriptive statistics were applied to describe the prevalence of symptomatic KOA and its distribution. Categorical data were presented as frequency (n) and percentage (%), and cross-tabulations with Chi-square tests were applied. Where data did not meet parametric assumptions, non-parametric *post-hoc* analyses (Z-scores from adjusted residuals) were used to detect significant group differences. Continuous variables such as age and BMI were presented as mean (standard deviation), as they were approximately normally distributed, supported by the Kolmogorov–Smirnov and Shapiro–Wilk tests. Cross-tabulations with Chi-square tests were also used to provide frequency estimates of the study population’s sociodemographic characteristics stratified by ethnicity. Binary logistic regression analysis, with age adjusted in the model, was conducted to identify predictors of poor KOA pain. Pain outcomes were categorized as either improved (pain scores reduced by ≥1.5 points) or poor (pain scores unchanged or increased by ≥1.5 points). The cut-off value of 1.5 points or 15% was based on recommendations for minimum clinically important improvement in chronic musculoskeletal pain, particularly in KOA populations (Salaffi et al., 2004). The Bonferroni *post-hoc* test was performed to identify differences in relevant analysis. The significance level was set at *p* < 0.05. Additionally, the Chi-square value ( $\chi^2$), odds ratio (OR), 95% confidence interval (CI), or Z-score was included to support relevant findings.

## Results

### Prevalence and distribution of symptomatic KOA (n = 3,529)

**Figure 1:**
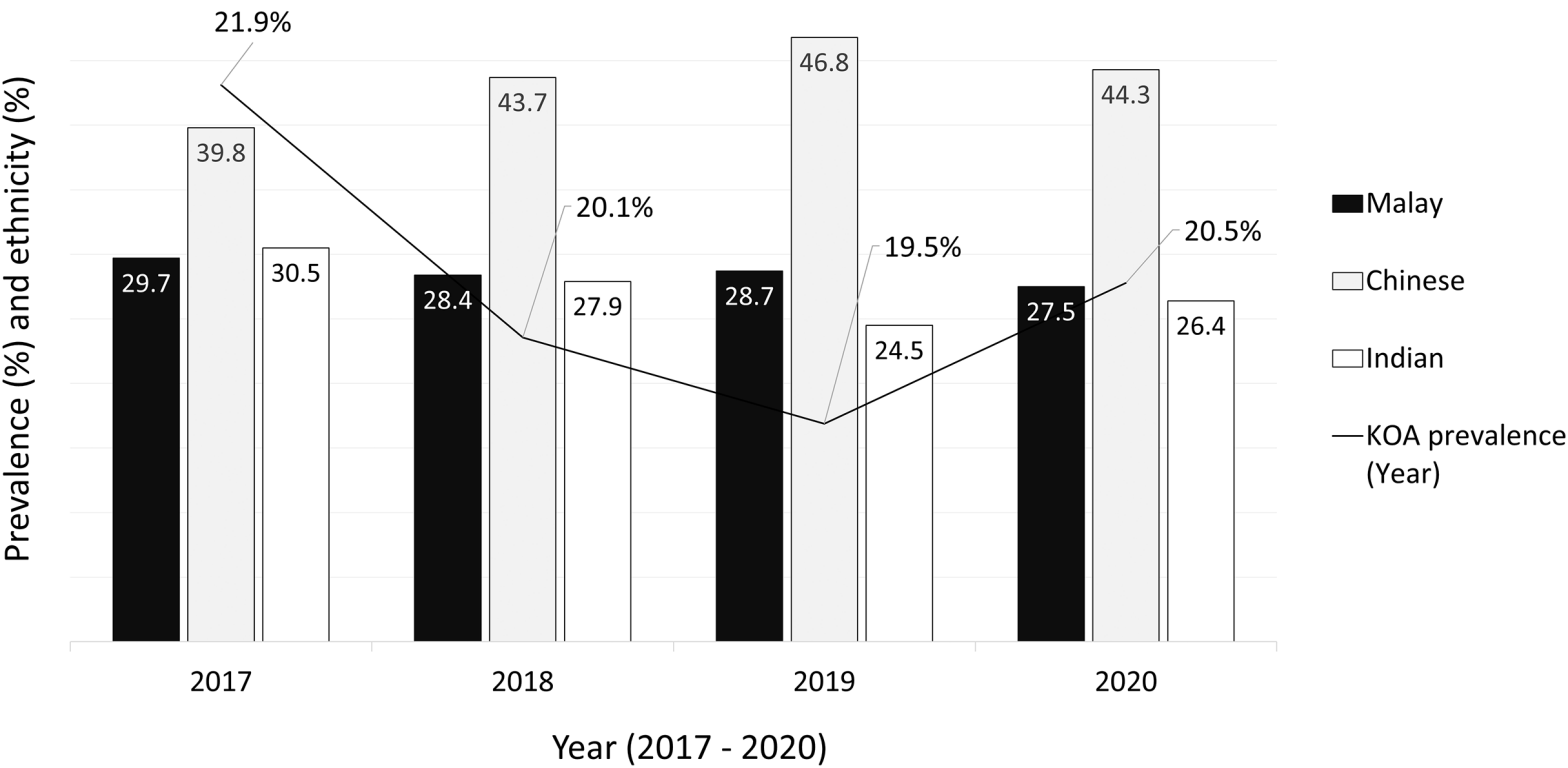
Prevalence of knee osteoarthritis and the ethnicity distribution from 2017 to 2020.

The UMMC Primary Care Clinics received a total of 17,253 new patients from January 2017 to December 2020, regardless of their diagnoses (Fig. 1). Among these patients, 3,529 (20.5%) were diagnosed with symptomatic KOA based on the American College of Rheumatology criteria, recorded in the ICD-10 coding system in the clinic records. Of these, 72.1% were female (*n* = 2,546, mean age 66.4 ± 11.5 years) and 27.9% were male (*n* = 983, mean age of 65.7 ± 12.9 years). The prevalence of KOA increased with age, with 6.9% of patients under the age of 44, 32.9% between 45 and 64, and 60.2% aged 65 years or older. In this clinical cohort, Chinese patients represented the highest proportion of individuals diagnosed with KOA (41.4%), followed by Malays (28.9%), Indians (27.6%), and others (2.1%).

### Characteristics of the symptomatic KOA population (n = 600)

Of the 3,529 patients newly diagnosed with symptomatic KOA, 600 medical records were randomly selected (stratified by ethnicity) and retrieved. Differences between ethnic groups were evaluated using one-way ANOVA for continuous variables (age) and Chi-square tests for categorical variables. *Post-hoc* analyses were conducted to identify specific group differences where significant *p*-values were found (*p* < 0.05). Significant differences were observed among ethnic groups in age, educational background, financial status, BMI, physical activity levels, cigarette smoking, and alcohol consumption (Table 1). Specifically, *post-hoc* analysis revealed the following significant ethnic differences: (i) age: Chinese patients (mean = 68 years) were significantly older than both Malay (mean = 60 years) and Indian (mean = 61 years) (*F* = 17.86, *p* < 0.001); (ii) BMI: 83.0% of Chinese patients were overweight compared to Malays (65.9%) and Indians (64.4%) (*Z* = 4.2, *p* = 0.021); (iii) physical activity: 43.0% of Chinese engaged in regular physical activity compared to Malays (22.2%) and Indians (20.0%) (*Z* = 4.6, *p* = 0.011); (iv) alcohol consumption: Chinese patients had the highest rates (62.9%) compared to Indians (30.4%) and Malays (1.5%) (*Z* = 6.6, *p* < 0.001). Conversely, Malay (73.3%) patients had a significantly higher percentage with tertiary education compared to both Chinese (28.9%) and Indian (34.1%) (*Z* = 3.7, *p* < 0.001). While Indian patients demonstrated (i) a higher proportion of financially aided individuals (33.3%) compared to Malays (18.5%) and Chinese (11.1%) (*Z* = 3.1, *p* = 0.001), and (ii) a higher percentage of smokers (28.9%) compared to Malays (17.8%) and Chinese (14.8%) (*Z* = 4.2, *p* = 0.027). Overall, significant sex differences were noted across ethnicities, with males exhibiting higher rates of cigarette smoking and alcohol consumption than females by 40.4% and 20.8%, respectively (*p* < 0.001).

**Table 1:** Sociodemographic characteristics of the study population stratified by ethnicity (*n* = 600).

Variables	Malay (*n* = 200) *n* (%)	Chinese (*n* = 200) *n* (%)	Indian (*n* = 200) *n* (%)	χ^2^-or *F*-value	*p*-value
Age, years: mean (SD)	60 (12)	68 (11)^a^	61 (13)	*F* = 17.86	0.001^b^
Age groups					
18–44	24 (11.9)	6 (3.0)	16.2 (8.1)	χ^2^ = 27.81	0.001^b^
45–64	101 (50.4)	61 (30.4)	101 (50.4)		
≥65	76 (37.8)	133 (66.7)^a^	83 (41.5)		
Sex					
Male	81 (40.7)	77 (38.5)	99 (49.7)	χ^2^ = 3.79	0.150
Female	119 (59.3)	123 (61.5)	101 (50.4)		
Education background					
Tertiary	147 (73.3)^a^	58 (28.9)	68 (34.1)	χ^2^ = 42.08	0.001^b^
Primary-secondary	53 (26.7)	142 (71.1)	131 (65.9)		
Financial status					
Unaided	163 (81.5)	178 (88.9)	133 (66.7)	χ^2^ = 19.92	0.001^b^
Aided	37 (18.5)	22 (11.1)	67 (33.3)^a^		
Body mass index					
≤22.9 normal	68 (34.1)	34 (17.0)	71 (35.6)	χ^2^ = 13.76	0.001^b^
≥23 overweight	132 (65.9)	166 (83.0)^a^	129 (64.4)		
Physical activity level					
Active	44 (22.2)	86 (43.0)^a^	40 (20.0)	χ^2^ = 21.85	0.001^b^
Inactivity/sedentary	155 (77.8)	114 (57.0)	160 (80.0)		
Cigarette smoking					
No	164 (82.2)	170 (85.2)	146 (73.3)	χ^2^ = 9.00	0.011^b^
Yes	36 (17.8)	30 (14.8)	58 (28.9)^a^		
Alcohol consumption					
No	197 (98.5)	74 (37.1)	139 (69.6)	χ^2^ = 106.66	0.001^b^
Yes	3 (1.5)	126 (62.9)^a^	61 (30.4)		

**Notes:**

Values are presented as *n* (%) within ethnicity apart from age as mean (SD). All variables were analyzed by cross-tabulations with the Chi-square test except for age (one-way ANOVA).

a Data with significant ethnic differences based on *post-hoc* analysis (*Z* > 1.96; *p* < 0.005).

b Statistically significant, *p* < 0.05.

Approximately 71.1% (*n* = 427) of the study population were diagnosed with KOA using radiographic assessment and classified according to the Kellgren-Lawrence system (Table 2); 34.0% (*n* = 145) were mild, 46.9% (*n* = 200) moderate, and 19.1% (*n* = 82) severe KOA. No significant differences were observed in Kellgren-Lawrence grades across the three ethnic groups. Of the total patients, 479 (80.0%) were referred for physiotherapy. Chinese patients (27.4%) were significantly less likely to receive physiotherapy compared with Malay (14.8%) and Indian patients (17.8%) (*p* = 0.001); however, no significant difference was observed between Malays and Indians. Rehabilitation adherence also varied significantly by ethnicity (*p* = 0.012). *Post-hoc* analysis indicated that non-adherence rates were significantly higher among Indians (75.0%) compared to Chinese (67.1%) and Malays (58.2%); while Malays (41.8%) were significantly more likely to adhere to the rehabilitation programme compared to Chinese (32.9%) (*p* = 0.021). Across all ethnic groups, non-adherence rates were 63.2% among patients with primary or secondary education (*p* = 0.006) and 67.1% among those receiving financial aid (*p* = 0.043). In contrast, only 30.4% of patients with tertiary education were non-adherent, and 41.9% of patients with unaided financial status demonstrated non-adherence, indicating that lower education and financial hardship were consistently associated with poorer rehabilitation adherence. Based on available knee pain score changes, 48.1% of the overall patients reported improvements, with at least a 1.5-point reduction in pain scores. *Post-hoc* analysis revealed that Malay patients (59.3%) experienced significantly higher percentage of improvements in knee pain compared to Chinese (40.7%) and Indian patients (44.4%) (*p* = 0.027), with no significant difference between Chinese and Indian groups.

**Table 2:** Disease severity and rehabilitation variables of the study population stratified by ethnicity (*n* = 600).

Variables	Malay (*n* = 200) *n* (%)	Chinese (*n* = 200) *n* (%)	Indian (*n* = 200) *n* (%)	χ^2^-value	*p*-value
Knee X-ray					
Yes	164 (82.2)	114 (57.0)^a^	149 (74.1)	χ^2^ = 13.03	0.001^b^
No	36 (17.8)	86 (42.9)	52 (25.9)		
Kellgren-Lawrence classification (*n* = 427)					
Grade ≤ 2 mild	68 (41.5)	31 (27.2)	46 (30.9)	χ^2^ = 8.72	0.069
Grade 3 moderate	65 (39.6)	59 (51.8)	75 (50.3)		
Grade 4 severe	31 (18.9)	24 (21.1)	27 (18.1)		
Physiotherapy					
No	30 (14.8)	55 (27.4)	36 (17.8)	χ^2^ = 8.23	0.016^b^
Yes	170 (85.2)	145 (72.6)^a^	164 (82.2)		
Rehabilitation (*n* = 479)					
Adhered	71 (41.8)^a^	46 (32.9)	41 (25.0)	χ^2^ = 12.76	0.012^b^
Non-adhered	99 (58.2)	94 (67.1)	123 (75.0)^a^		
Changes in knee pain					
Improved pain	118 (59.3)^a^	81 (40.7)	89 (44.4)	χ^2^ = 10.97	0.027^b^
Unchanged	15 (7.5)	18 (8.9)	15 (7.4)		
Worsened pain	67 (33.3)	101 (50.4)	96 (48.1)		

**Notes:**

Values are presented as frequency (n) and percentage (%) within ethnicity. All variables were analyzed by cross-tabulations with the Chi-square test.

a Data with significant ethnic differences based on *post-hoc* analysis (*Z* > 1.96; *p* < 0.005).

b Statistically significant, *p* < 0.05.

### Predictors of poor KOA pain outcomes

Across all ethnicities, a sedentary or inactive lifestyle was a significant predictor of poor KOA pain outcomes (Table 3); Malay (adjusted OR: 3.09; 95% CI [1.08–8.83]; *p* = 0.035), Chinese (adjusted OR: 3.62; 95% CI [1.71–7.66]; *p* = 0.001), and Indian (adjusted OR: 3.43; 95% CI [1.31–8.96]; *p* = 0.012). Among those with primary-secondary education backgrounds, the odds of having poor KOA pain outcomes were seven times higher for Malays (95% CI [2.60–20.98]; *p* < 0.0001) and four times higher for Indians (95% CI [1.36–8.24]; *p* = 0.009). In addition, regular alcohol consumption among Chinese (95% CI [1.50–9.75]; *p* = 0.005) and Indian (95% CI [1.56–11.95]; *p* = 0.005) increased the odds by approximately four-fold compared to those who did not drink.

**Table 3:** Ethnic comparison of adjusted odds ratios with 95% confidence intervals for poor KOA pain outcomes based on socioeconomic variables (adjusted for age).

Variables (Reference category)	Adjusted odds ratio of poor KOA pain outcomes (95% confidence interval); *p*-value		
	Malay (*n* = 200)	Chinese (*n* = 200)	Indian (*n* = 200)
Education (Tertiary)	–	–	–
Primary-secondary	7.39 (2.60–20.98); *p* < 0.001^a^	1.30 (0.42–4.00); *p* = 0.650	3.34 (1.36–8.24); *p* = 0.009^a^
Financial (Unaided)	–	–	–
Aided	2.35 (0.79–6.99); *p* = 0.123	0.73 (0.28–1.87); *p* = 0.508	1.46 (0.68–3.13); *p* = 0.334
BMI (≤22.9 normal)	–	–	–
≥23 overweight to obese	0.83 (0.38–1.79); *p* = 0.827	0.88 (0.33–2.037); *p* = 0.804	1.08 (0.52–2.25); *p* = 0.834
Cigarette smoking (No)	–	–	–
Yes	0.31 (0.10–0.97); *p* = 0.044^a^	1.22 (0.45–3.29); *p* = 0.695	1.18 (0.54–2.57); *p* = 0.683
Alcohol intake (No)	–	–	–
Yes	0 (0–0); *p* = 0.999	3.82 (1.50–9.75); *p* = 0.005^a^	4.32 (1.56–11.95); *p* = 0.005^a^
Physical activity (Active)	–	–	–
Inactivity/sedentary	3.09 (1.08–8.83); *p* = 0.035^a^	3.62 (1.71–7.66); *p* = 0.001^a^	3.43 (1.31–8.96); *p* = 0.012^a^

**Notes:**

BMI, body mass index; KOA, knee osteoarthritis. The odds ratios in this table have been adjusted for age, ensuring the relationships between socioeconomic variables and KOA pain outcomes are not influenced by age.

a Statistically significant, *p* < 0.05.

Based on disease severity, the odds of poor KOA pain outcomes were five to thirteen times higher among those categorised as having severe KOA than mild: Malay (adjusted OR: 5.47, 95% CI [1.64–18.27]; *p* = 0.006), Chinese (adjusted OR: 8.25, 95% CI [1.79–38.01]; *p* = 0.007) and Indian (adjusted OR: 13.09, 95% CI [2.51–8.18]; *p* = 0.002) (Table 4). Meanwhile, the odds of having poor KOA pain outcomes among those who did not receive physiotherapy were nine times more likely for Malays (95% CI [2.27–33.10]; *p* = 0.002) and three times higher for Chinese (95% CI [1.22–7.40]; *p* = 0.017) compared to those who received physiotherapy treatment. The odds also increased by up to seventeen-fold among patients who did not adhere to rehabilitation compared to those who adhered; Malay (95% CI [5.18–57.03]; *p* < 0.001) and Chinese (95% CI [4.90–56.10]; *p* < 0.001).

**Table 4:** Ethnic comparison of adjusted odds ratio with 95% confidence interval for poor KOA pain outcomes based on disease severity and rehabilitation (adjusted for age).

Variables (Reference category)	Adjusted odds ratio of poor KOA pain outcomes (95% confidence interval); *p*-value		
	Malay (*n* = 200)	Chinese (*n* = 200)	Indian (*n* = 200)
K-L classification (G1/2 mild)	–	–	–
G3 moderate	2.98 (1.11–7.96); *p* = 0.030^a^	4.60 (1.38–15.32); *p* = 0.013^a^	1.13 (0.43–2.96); *p* = 0.799
G4 severe	5.47 (1.64–18.27); *p* = 0.006^a^	8.25 (1.79–38.01); *p* = 0.007^a^	13.09 (2.51–8.18); *p* = 0.002^a^
Physiotherapy (Yes)	–	–	–
No	8.67 (2.27–33.10); *p* = 0.002^a^	3.00 (1.22–7.40); *p* = 0.017^a^	1.08 (0.39–3.02); *p* = 0.878
Rehabilitation (Adhered)	–	–	–
Non-adhered	17.20 (5.18–57.03); *p* < 0.001^a^	16.57 (4.90–56.10); *p* < 0.001^a^	3.46 (1.47–8.14); *p* = 0.004^a^

**Notes:**

K-L, Kellgren-Lawrence; KOA, knee osteoarthritis. The odds ratios in this table have been adjusted for age, ensuring that the relationships between disease severity, rehabilitation, and KOA pain outcomes are not influenced by age.

a Statistically significant, *p* < 0.05.

## Discussion

This study aimed to examine the prevalence of symptomatic KOA and the influence of associated sociodemographic factors, and to identify predictors of KOA pain outcomes among the multi-ethnic Malaysian urban population. The findings suggest that the prevalence of symptomatic KOA among the urban population in Malaysia is high, and sociodemographic factors significantly influence the distributions; both non-modifiable (ethnicity, age, sex, education and financial) and modifiable (BMI, smoking, alcohol and physical activity). It is worth noting that, to ensure conceptual clarity and to avoid multicollinearity or overadjustment, socioeconomic and lifestyle predictors (Table 3) and clinical or rehabilitation-related predictors (Table 4) were analyzed in separate age-adjusted models, as these domains represent distinct determinants within the KOA outcome pathway. In this study, ethnicity was examined as a sociocultural and contextual determinant reflecting differences in lifestyle practices, health behaviours, and access to healthcare, rather than as a biological construct. This approach was guided by the study hypothesis and the Malaysian urban context, where ethnic background is closely intertwined with modifiable risk factors relevant to KOA prevention and rehabilitation.

### Study rationale and the role of ethnicity in KOA within urban Malaysia

The prevalence of KOA in Malaysia is comparable to other countries; for example, the United States at 19.0% (Wallace et al., 2017), China at 17.4% (Liu et al., 2016), and India at 26.8% (Venkatachalam et al., 2018). These statistics indicate that KOA is highly prevalent worldwide and should be seen as a serious threat (Courties et al., 2024), considering the disabling impact on individuals (Ahmad et al., 2018) and the healthcare burden of the disease (Kamsan et al., 2020; Munoz Laguna et al., 2023). Additionally, ethnicity in Malaysia was found to influence the prevalence distributions, which has also been observed in other nations (Callahan et al., 2021; Ji et al., 2023). In this study, Chinese, followed by Malay and Indian, was the prevalence order for ethnicity among symptomatic KOA. This distribution can be anticipated, as the recent literature reported that the Chinese population in Malaysia demonstrated a higher level of health awareness and was more likely to undergo medical screening than Malays or Indians (Cheah & Meltzer, 2020). However, according to Mat et al. (2019), the highest prevalence of KOA was observed among Malays, followed by Indians and then Chinese, based on data spanning from 2013 to 2015. This discrepancy could be attributed to differences in their study criteria, which included self-reported symptoms and focused on older adults (Mat et al., 2019). It is important to note that their study sample, drawn from the Malaysian Elders Longitudinal Research (MELoR), was obtained through stratified random sampling of electoral constituencies. However, this method may not accurately represent the urban population, as it encompassed individuals from areas outside the Klang Valley.

Furthermore, the variance in ethnic prevalence of KOA, with Mat et al. (2019) identifying a higher prevalence among ethnic Malays, contrasts with our findings where Chinese ethnicity predominated. This discrepancy could be attributed to the urban-rural distribution of ethnic groups, as Chinese individuals are predominantly located in urban areas, while Malays and Indians are more dominant in rural regions (Peng & Li, 2022). Thus, the standard clinical diagnostic methods (ACR criteria) and the heterogeneous sample criteria employed in this study are believed to provide information of higher validity and reliability (Plotnikoff et al., 2015) regarding KOA distribution in the urban Malaysian population.

### Non-modifiable factors affecting KOA pain outcomes

Other non-modifiable factors identified were age (≥65 years), female sex, educational and financial status associated with the prevalence of KOA in Malaysia, comparable with reports from other countries (Courties et al., 2024; Szilagyi et al., 2023). Increasing age is highly associated with KOA prevalence due to; (i) generalised decline in muscles mass and strength of the quadriceps and hamstring as knee joint stabilisers (Zhang et al., 2020), and (ii) increased levels of reactive oxygen species which inhibit signalling pathways that sustain the extracellular matrix, leading to cartilage degeneration (Chow & Chin, 2020). Secondly, KOA is reported to be sexually dimorphic, with females being twice as likely to develop the disease as males (Szilagyi et al., 2023), which was attributed to post-menopausal hormonal changes (oestrogen deficiency and low sensitivity to hormonal stimuli) and genetic coding (inflammatory mediators and hormonal receptors) that are involves in cartilage metabolism (Contartese et al., 2020).

In addition, 54.5% of patients with KOA in this study were from primary-secondary education backgrounds and 20.9% with low financial status (financially aided), both of which were highest among Indians. Of these, about 53.5% were non-adherent to the rehabilitation session, and 51.9% recorded poor KOA pain outcomes. Based on the previous study by Mat et al. (2019), lower educational levels and lower financial status were significantly associated with knee pain (Mat et al., 2019); these observations are aligned with this study, although the population under study was different. Thus, these two factors (education and financial status) play a major role in both geographical demographics, whether urban or rural. It is postulated that despite the highest prevalence among the Chinese, they have a higher educational background and are more financially stable, hence, better rehabilitation adherence with favourable KOA pain outcomes. In agreement, Lee et al. (2021) reported a similar association between educational and financial background and KOA-related variables. In general, the low education-financial population is associated with intensive physical activity endured in life which stresses the knee (Wang et al., 2020) and defaulted treatment due to costly long-term rehabilitation which precipitates the development and adverse progression of KOA (Lee et al., 2021). Beyond this, differences in education are also likely to influence the nature of employment, with individuals of lower educational attainment often working in physically demanding jobs that increase mechanical stress on the knees, while those with higher education tend to access less physically strenuous occupations with better health awareness (Callahan et al., 2011). Our findings imply that, relative to educational background, financial status plays a more significant role in affecting KOA distributions and its related outcomes; stable financial status leads to better treatment access and adherence, with less KOA pain.

### Modifiable factors affecting KOA pain outcomes

The modifiable factors associated with symptomatic KOA were BMI, cigarette smoking, alcohol consumption, and physical inactivity. Notably, Mat et al. (2019), in their analysis of a mixed urban and rural population, did not report on cigarette smoking, alcohol consumption, or physical inactivity. These aspects are novel findings uncovered in this study, particularly when examined through the lens of ethnic-specific differences that inform targeted prevention strategies. While for BMI, our findings are in agreement with Mat et al. (2019), suggesting that BMI is a predominant factor contributing to KOA, regardless of whether the population is urban or rural. Obesity stands out as a significant factor in both the development and progression of KOA, as widely discussed by previous studies (Lv et al., 2024; Mündermann et al., 2024). Excessive weight gain increases mechanical loading to the synovial joint and could damage the knee joint’s articular cartilage (Mündermann et al., 2024). Evidently, the probability of developing KOA increased by 35.0% for every 5 kg/m^2^ increment in BMI score regardless of ethnic background (Zheng & Chen, 2015). This finding underscores the critical role of BMI as a common risk factor contributing to KOA in both urban and rural populations, highlighting the importance of addressing and managing obesity to reduce the burden of this debilitating condition across diverse demographics.

The present study found that 20.5% of patients with KOA were smokers; this low rate is expected considering most patients were female (72.1%), whilst cigarette smoking is common among males (Lim et al., 2018). Specifically, based on (i) sex: 44.7% and 4.3% of male and female patients respectively were smokers, respectively, and (ii) ethnicity: Indian (28.9% of smokers) compared to Malay (17.8%) or Chinese (14.8%). Despite the lack of significant difference between smokers and non-smokers in radiographic KOA severity or knee pain outcomes in this study, smoking undoubtedly compromises patients with other health risks, such as hypertension and diabetes mellitus, which are known to increase the risk of adverse KOA progression (Seow et al., 2024). Thus, smoking cessation initiatives targeting Indian patients, particularly men, are essential as an ethnic-specific prevention strategy to reduce overall disease burden.

Alcohol consumption increases susceptibility to the development and progression of KOA (Joseph et al., 2019; Kang et al., 2020). Alcohol consumption is correlated with radiological knee joint degeneration as indicated by defective cartilage and meniscus morphology (Kang et al., 2020). Incidentally, this factor is highest among the Chinese (62.9%) which corroborates with the ethnic prevalence findings. In agreement, previous literature reported that around 70.0% of Chinese, 42.0% of Indians and 11.0% of Malays were drinkers and these rates are further supported by higher financial status (Mutalip et al., 2014). This polarity is due to cultural and religious prohibitions, as most Malays are Muslim (Arshad, Rozali & Kamaruzzaman, 2012; Mutalip et al., 2014). Accordingly, reducing alcohol intake among Chinese patients should be emphasised as an ethnic-specific prevention strategy.

In addition, physical inactivity emerged as another important modifiable factor, with 71.6% of patients identified as physically inactive, particularly among Malays, Indians, and those aged ≥65 years. Malays recorded the highest rates of inactivity in this cohort. Sedentary lifestyles, whether due to cultural habits, occupational patterns, or limited engagement in structured exercise, exacerbate obesity-related risks and accelerate functional decline in KOA (Kraus et al., 2019; Seow et al., 2024; Zulfarina et al., 2022). Many elderly patients reported restricting their physical activity in the belief that this would reduce KOA symptoms, particularly knee pain, reflecting fear-avoidance behaviours (Larsson et al., 2016). However, there is no evidence to suggest that physical activity hastens KOA progression (Kraus et al., 2019). On the contrary, this study found a significantly greater decrease in KOA pain among those who were physically active (68.8% improved) compared to inactive patients (46.2%). This underscores the importance of culturally tailored strategies to promote physical activity, particularly among Malays, which may play a dual role in controlling BMI and improving musculoskeletal function, while also addressing misconceptions and fear-avoidance beliefs in older adults.

## Strengths and limitations

To our knowledge, this is the first study in Malaysia reporting the prevalence based on a definite KOA diagnosis according to the ACR criteria from a heterogeneous sample. The specific focus on urban populations compared to rural settings is also significant for understanding disease epidemiology, with urban data likely to be more reliable given larger sample sizes and more consistent access to healthcare services. Nevertheless, several limitations should be acknowledged. First, the findings may not be generalisable to all urban populations in Malaysia, as the study was conducted in a single centre within the Klang Valley. Replication in other major urban areas such as Georgetown or Johor Bahru would be important to validate the consistency of these findings. Second, the use of medical records data introduces certain constraints. Although diagnoses were based on standardised ACR criteria and documented within the ICD-10 system, reliance on existing records carries an inherent risk of coding or recording variability. Efforts were made to mitigate this through cross-validation with the Medical Records Department and consultant review, although some residual risk cannot be fully excluded. Third, although 200 records were available per subgroup, these analyses may still be underpowered to detect smaller effect sizes and should therefore be interpreted as exploratory.

Fourth, multivariable logistic regression including multiple predictors was not feasible due to the modest sample size and subgroup distribution, which could have led to overfitting and unstable estimates. For this reason, predictors were analysed in separate age-adjusted models, and future studies with larger multicentre datasets should apply full multivariable regression analyses with sample size calculations tailored to subgroup comparisons to strengthen reproducibility and external validity. An additional methodological consideration relates to the use of ethnicity-stratified random sampling within a cross-sectional design. While stratification ensured adequate representation of the three major ethnic groups for subgroup analyses, the resulting sample proportions do not fully mirror the true ethnic distribution of the underlying clinic population. Consequently, certain sociodemographic or lifestyle risk factors may be over-or under-represented within specific ethnic strata. These subgroup analyses were therefore interpreted as exploratory, with the primary inferences of this study drawn from the overall cohort.

## Conclusion

This study revealed a high prevalence of symptomatic KOA among urban Malaysians, predominantly affecting individuals aged 65 or older, females, and those who are overweight or obese. Ethnicity played a significant role, with Chinese exhibiting the highest prevalence, followed by Malays and Indians. Ethnic differences also extended to socioeconomic factors, such as low financial status, most common among Indians, which was associated with rehabilitation non-adherence and poor KOA pain outcomes. Alcohol consumption, predominantly among Chinese, was linked to severe degenerative knee joint changes and unfavourable KOA pain outcomes. It is worth highlighting that prior research, while similar in nature, did not specifically focus on the urban population or provide data on cigarette smoking, alcohol consumption, and physical inactivity; which are new findings uncovered by the current study within the context of ethnic differences. In light of these findings, this study underscores the necessity for ethnic-specific prevention and management strategies, like promoting physical activity for Malays, encouraging smoking cessation for Indians, and reducing alcohol intake for Chinese patients, to improve KOA outcomes.

## Supplemental Information

10.7717/peerj.20911/supp-1 Supplemental Information 1 Odds of poor KOA pain outcomes.

10.7717/peerj.20911/supp-2 Supplemental Information 2 Data.

10.7717/peerj.20911/supp-3 Supplemental Information 3 Clinical Research Form.

10.7717/peerj.20911/supp-4 Supplemental Information 4 SPSS statistical output.

10.7717/peerj.20911/supp-5 Supplemental Information 5 STROBE checklist.

## Additional Information and Declarations

### Data Availability

The following information was supplied regarding data availability:

The raw data are available in the Supplemental File.
