# Supplementary material for: Ethnic impact on knee osteoarthritis pain predictors in an urban Malaysian population: a retrospective study
Source: PeerJ. 2026 Mar 13;14:e20911. doi: 10.7717/peerj.20911 (PMC12990897; doi:10.7717/peerj.20911)
Supplement: Supplemental Information 3 [file peerj-14-20911-s003.pdf]

Appendix 1. Clinical Research Form.

RUKA CLINICAL RESEARCH FORM : KNEE OSTEOARTHRITIS

Incidence of symptomatic knee osteoarthritis and the related factors among patients visiting primary clinics at the University Malaya Medical Centre, Malaysia

* Required

1. Coded ID: *

2. Nationality: *

Mark only one oval.

- Malaysian
- Non-Malaysian

3. Year of visit: *

Mark only one oval.

- 2017
- 2018
- 2019

Part 2: Socio-Demographic Information

4. Age (years): *

5. Age group ranges: *

Mark only one oval.

- < 18 : Adolescent
- 18 – 35 : Young adult
- 36 – 55 : Middle-aged adult
- > 55 : Older adult

6. Gender: *

Mark only one oval.

- Male
- Female

7. Ethnicity: *

Mark only one oval.

- Malay
- Chinese
- Indian
- Others

8. Marital status: *

Mark only one oval.

- Unknown
- Single
- Married
- Widowed

9. Educational level: *

Mark only one oval.

- Unknown
- No formal education
- Primary & Secondary education
- Certificate, University & Higher education

10. Financial status: *

Mark only one oval.

- Unknown
- Unstable financial: job insecurity/ welfare assistance/ financial issues or etc.
- Stable financial: working with steady income/ no financial issues reported or etc.

11. Body mass index, BMI (kg/m2): *

Mark only one oval.

- Unknown
- <18.5 : underweight
- 18.5–24.9 : normal weight
- 25.0–29.9 : overweight
- 30.0–34.9 : class I obesity
- 35.0–39.9 : class II obesity
- > 40 : morbid obesity

12. Additional information (demographic)?

____

Part 3: Current History (Knee Osteoarthritis)

13. Method of KOA diagnosis: *

Mark only one oval.

- ( ) Clinical criteria alone
- ( ) Radiographic investigation (Clinical + Radiographic)
- ( ) Blood investigation (Clinical + Blood test)
- ( ) All (Clinical + Radiographic + Blood investigation)

14. Diagnostic radiology/ blood investigation performed: *

Check all that apply.

- [ ] NO (no radiographic or blood investigation performed)
- [ ] YES;
- [ ] X-ray
- [ ] Magnetic resonance imaging (MRI)
- [ ] CT Scan
- [ ] Ultrasound
- [ ] Biomarkers analysis

15. Radiographic Classification of KOA (Kellgren-Lawrence Classification System): *

Mark only one oval.

- ( ) Unknown (no radiographic investigation performed)
- ( ) Grade 0 - no presence of OA.
- ( ) Grade 1 - doubtful narrowing of the joint space with possible osteophyte formation.
- ( ) Grade 2 - narrowing of the joint space with definite osteophyte formation.
- ( ) Grade 3 - definite narrowing of joint space, moderate osteophyte formation, some sclerosis, and possible deformity of bony end.
- ( ) Grade 4 - large osteophyte formation, severe narrowing of the joint space with marked sclerosis, and definite deformity of bone end.

16. Affected side due to KOA: *

Mark only one oval.

- ( ) Right
- ( ) Left
- ( ) Bilateral

17. Affected knee compartment(s) due to KOA: *

Check all that apply.

- [ ] Unknown (no information available)
- [ ] Lateral tibiofemoral compartment
- [ ] Medial tibiofemoral compartment
- [ ] Patellofemoral compartment

18. Any other joint(s) affected by osteoarthritis or any other form of arthritis? If the answer is YES, please provide details: *

Check all that apply.

- [ ] NO
- [ ] YES;
- [ ] Shoulder
- [ ] Elbow
- [ ] Hand
- [ ] Spine
- [ ] Hip
- [ ] Ankle
- [ ] Toes
- [ ] Others

19. Possible causes of knee osteoarthritis: *

Mark only one oval.

- ( ) Primary KOA/ Idiopathic
- ( ) Secondary KOA

20. Identified KOA risk factors: *

Check all that apply.

- [ ] Ageing
- [ ] Obesity/ Overweight
- [ ] Malposition (Varus/Valgus)
- [ ] Congenital abnormalities or malformation of the limb
- [ ] Post-surgical
- [ ] Traumatic
- [ ] Sport injury related (previous injury or procedures)
- [ ] Ligamentous injury
- [ ] Metabolic disease; hypertension, diabetes mellitus, dyslipidemia
- [ ] Other form of arthritis: gouty, rheumatoid, degenerative disc disease and etc.

21. Baseline signs and symptoms: *

Check all that apply.

- [ ] Knee pain at rest
- [ ] Knee pain during movement
- [ ] Limited knee movement
- [ ] Knee stiffness
- [ ] Knee swelling/ effussion
- [ ] Crepitus
- [ ] Limited flexibility
- [ ] Instability
- [ ] Reduced lower extremity muscle strength (weakness)
- [ ] Gait abnormalities
- [ ] Balance issues

22. Impact of knee osteoarthritis: *

Check all that apply.

- [ ] ADL limitations; eating, bathing, dressing, toileting, mobility, and grooming
- [ ] IADL limitations; more complex sets of skills-using the telephone, shopping, preparing meals, housekeeping, using transportation, taking medication and managing finances.
- [ ] Need assistance with ADL or IADL
- [ ] Job limitations
- [ ] Unknown limitations

23. Use of mobility aids? *

Mark only one oval.

- ( ) No or Unknown (no information available)
- ( ) Yes

24. Use of any mobility aids: If the answer is YES, please provide details: *

Check all that apply.

- [ ] NO or unknown (no information available)
- [ ] YES;
- [ ] Knee brace
- [ ] Single point stick
- [ ] Tripod/ Quadripod
- [ ] Walking frame
- [ ] Wheelchair
- [ ] Others

25. Pharmacological management of KOA: *

Check all that apply.

- [ ] No or unknown (no information available)
- [ ] Yes

26. Pharmacological management of KOA: If the answer is YES, please provide details: *

Check all that apply.

- [ ] NO or unknown (no information available)
- [ ] YES;
- [ ] NSAIDs: aspirin, celexocib (celebrex), diclofenic, ibuprofen
- [ ] Glucosamine and Chondroitin Sulfate
- [ ] Acetaminophen
- [ ] Injections of corticosteroids or hyaluronic acid
- [ ] Others

27. Any previous surgical management for KOA? *

Mark only one oval.

- ( ) No or unknown (no information available)
- ( ) Yes

28. Additional information (knee osteoarthritis):

____

Part 4 : Comorbidity, Medical & Surgical History

29. Comorbidity and medical history: *

Check all that apply.

- [ ] NKMI (No known medical illness)
- [ ] Obesity
- [ ] Diabetes Mellitus
- [ ] Hypertension
- [ ] Dyslipidemia
- [ ] Hypercholesterolemia
- [ ] Osteoporosis
- [ ] Malignancy
- [ ] Blood disorders
- [ ] Respiratory disease
- [ ] Cardiovascular disease
- [ ] Neurological disease
- [ ] Renal disease
- [ ] Autoimmune disease
- [ ] Cognitive impairment
- [ ] Other form of arthritis: gouty, rheumatoid and etc
- [ ] Others

30. Previous surgical history: *

Mark only one oval.

- Unknown (No information available)
- No
- Yes

31. Additional information (comorbidity, medical and surgical history)

Part 5: Social History

32. Smoking status: *

Mark only one oval.

- Unknown (No information available)
- Never smoker.
- Current smoker
- Ex-smoker

33. Alcohol consumption: *

Mark only one oval.

- Unknown (No information available)
- No
- Yes

34. Physical activity status: *

Mark only one oval.

- Unknown (No information available)
- Sedentary lifestyle (no regular physical activity or exercise)
- Active lifestyle (regular physical activity or exercise; gardening, exercise, sport participation and etc.)

Part 6: Physiotherapy & Treatment Response

35. Referral for physiotherapy management: *

Mark only one oval.

- No (no referral/ no record of physiotherapy treatment/ no information available)
- Yes

36. Referred physiotherapy service: *

Mark only one oval.

- ( ) No (no referral/ no record of physiotherapy treatment/ no information available)
- ( ) Rehabilitation Services UMMC
- ( ) Others: Public or private services

37. Method of physiotherapy management delivery: *

Check all that apply.

- [ ] No (no referral/ no record of rehabilitation/ no information available)
- [ ] Supervised session
- [ ] Home-based exercise program

38. Received physiotherapy intervention(s): *

Check all that apply.

- [ ] No (no referral/ no record of rehabilitation/ no information available)
- [ ] Therapeutic exercise: stretching, strengthening, mobilizing and etc.
- [ ] Weight-bearing exercise
- [ ] Non-weight bearing exercise
- [ ] Manual therapy: joint mobilization, soft tissue manipulation and etc.
- [ ] Electro-modalities: TENS, laser therapy, TENS and etc.
- [ ] Heat therapy (hot pack/ cold pack)
- [ ] Taping techniques
- [ ] Knee support
- [ ] Aerobic exercise
- [ ] Patient education
- [ ] Home exercise
- [ ] Hydrotherapy
- [ ] Weight management: advise/ program
- [ ] Cardiac Rehabilitation Program/ Chest physiotherapy
- [ ] Others

39. Frequency of visit (session per month): *

Mark only one oval.

- ( ) No (no referral/ no record of rehabilitation/ no information available)
- ( ) Only attended one session
- ( ) < 1 (per month)
- ( ) 1 - 2 (per month)
- ( ) 3 - 4 (per month)
- ( ) > 4 (per month)

40. Treatment response, BASELINE knee pain score: *

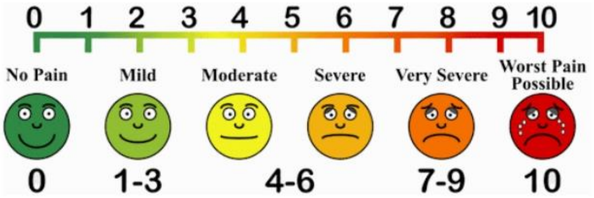

Mark only one oval.

- No (no referral/ no record of rehabilitation/ no information available)
- 0 (no pain)
- 1
- 2
- 3
- 4
- 5
- 6
- 7
- 8
- 9
- 10 (worst pain)

41. Treatment response, FOLLOW-UP knee pain score: *

Mark only one oval.

- No (no referral/ no record of rehabilitation/ no information available)
- 0 (no pain)
- 1
- 2
- 3
- 4
- 5
- 6
- 7
- 8
- 9
- 10 (worst pain)

42. Treatment response, knee pain score (s): *

Mark only one oval per row.

	No (no referral/ no record of rehabilitation/ no information available)	0 (no pain)	1	2	3	4	5	6	7	8	9	(w p
Baseline												
Follow-up												

43. Treatment response, BASELINE active knee flexion range: *

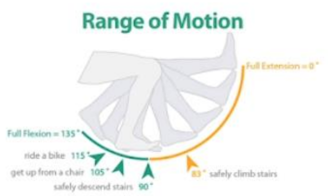

Mark only one oval.

- No (no referral/ no record of rehabilitation/ no information available)
- < 45 degree
- < 90 degree
- < 120 degree
- AFROM

44. Treatment response, FOLLOW-UP active knee flexion range: *

Mark only one oval.

- No (no referral/ no record of rehabilitation/ no information available)
- < 45 degree
- < 90 degree
- < 120 degree
- AFROM

45. Treatment response, active knee flexion range: *

Mark only one oval per row.

	No (no referral/ no record of rehabilitation/ no information available)	< 45 degree	< 90 degree	< 120 degree	AFROM
Baseline	⬭	⬭	⬭	⬭	⬭
Follow-up	⬭	⬭	⬭	⬭	⬭

46. Treatment response, BASELINE lower limb muscle strength: *

Mark only one oval.

- ⬭ No (no referral/ no record of rehabilitation/ no information available)
- ⬭ 0
- ⬭ 1
- ⬭ 2
- ⬭ 3
- ⬭ 4
- ⬭ 5

47. Treatment response, FOLLOW-UP lower limb muscle strength: *

Mark only one oval.

- ⬭ No (no referral/ no record of rehabilitation/ no information available)
- ⬭ 0
- ⬭ 1
- ⬭ 2
- ⬭ 3
- ⬭ 4
- ⬭ 5

48. Treatment response, lower limb muscle strength: *

Mark only one oval per row.

	No (no referral/ no record of rehabilitation/ no information available)	0	1	2	3	4	5
Baseline	⬭	⬭	⬭	⬭	⬭	⬭	⬭
Follow-up	⬭	⬭	⬭	⬭	⬭	⬭	⬭

49. KOA progression, comparison between baseline and latest follow-up status: *

Mark only one oval.

- No (no referral/ no record of rehabilitation/ no information available)
- Unchanged
- Improving
- Improved, discharge with self-management
- Worsening, but no surgical intervention performed
- Worsen, surgical intervention performed (TKR)

50. Overall assessment of patient compliance with physiotherapy/ rehabilitation: *

Mark only one oval.

- No (no referral/ no record of rehabilitation/ no information available)
- Lack of compliance/ adherence: only attend 1 session/ refuse for further exercise/ skipped appoinment regularly and etc
- Good compliance/ adherence: regular rehabilitation visits/ comply with HBE and etc

51. Additional information (physiotherapy and treatment response):

____

This content is neither created nor endorsed by Google.

Google Forms
